# Abundance of Early Functional HIV-Specific CD8^+^ T Cells Does Not Predict AIDS-Free Survival Time

**DOI:** 10.1371/journal.pone.0002745

**Published:** 2008-07-23

**Authors:** Ingrid M. M. Schellens, José A. M. Borghans, Christine A. Jansen, Iris M. De Cuyper, Ronald B. Geskus, Debbie van Baarle, Frank Miedema

**Affiliations:** 1 Department of Immunology, University Medical Center Utrecht, Utrecht, The Netherlands; 2 Theoretical Biology/Bioinformatics, Utrecht University, Utrecht, The Netherlands; 3 Department of Clinical Viro-Immunology, Sanquin Research and Landsteiner Laboratory of the Academic Medical Center, University of Amsterdam, Amsterdam, The Netherlands; 4 Department of Research, Cluster, Infectious Diseases, Health Service of Amsterdam, Amsterdam, The Netherlands; 5 Department of Clinical Epidemiology, Biostatistics and Bioinformatics, Academic Medical Center, University of Amsterdam, Amsterdam, The Netherlands; Federal University of Sao Paulo, Brazil

## Abstract

**Background:**

T-cell immunity is thought to play an important role in controlling HIV infection, and is a main target for HIV vaccine development. HIV-specific central memory CD8^+^ and CD4^+^ T cells producing IFNγ and IL-2 have been associated with control of viremia and are therefore hypothesized to be truly protective and determine subsequent clinical outcome. However, the cause-effect relationship between HIV-specific cellular immunity and disease progression is unknown. We investigated in a large prospective cohort study involving 96 individuals of the Amsterdam Cohort Studies with a known date of seroconversion whether the presence of cytokine-producing HIV-specific CD8^+^ T cells early in infection was associated with AIDS-free survival time.

**Methods and Findings:**

The number and percentage of IFNγ and IL-2 producing CD8^+^ T cells was measured after in vitro stimulation with an overlapping Gag-peptide pool in T cells sampled approximately one year after seroconversion. Kaplan-Meier survival analysis and Cox proportional hazard models showed that frequencies of cytokine-producing Gag-specific CD8^+^ T cells (IFNγ, IL-2 or both) shortly after seroconversion were neither associated with time to AIDS nor with the rate of CD4^+^ T-cell decline.

**Conclusions:**

These data show that high numbers of functional HIV-specific CD8^+^ T cells can be found early in HIV infection, irrespective of subsequent clinical outcome. The fact that both progressors and long-term non-progressors have abundant T cell immunity of the specificity associated with low viral load shortly after seroconversion suggests that the more rapid loss of T cell immunity observed in progressors may be a consequence rather than a cause of disease progression.

## Introduction

With an estimated 3 million human immunodeficiency virus (HIV)-related deaths each year, an effective treatment for HIV is urgently needed. Highly active antiretroviral therapy (HAART) results in significant suppression of HIV viral load, but is unable to eradicate the virus from the body, has severe side effects and may lead to resistance. An alternative option to prevent HIV infection and progression to acquired immunodeficiency syndrome (AIDS) might be therapeutic or prophylactic vaccination.

It is generally accepted that CD8^+^ T cells play an important role in controlling HIV replication. The fact that i) the decline in viral load in acute infection coincides with the peak in HIV-specific CD8^+^ T-cell numbers [1], [2], ii) depletion of CD8^+^ T cells in macaques results in higher viral load [3]–[5], and iii) escape mutations have frequently been observed in CTL epitopes [6]–[9], points to a role for these cells in the control of HIV infection. Therefore, an important line of HIV vaccine development focuses on the generation of cell-mediated immune responses. Although prevention of HIV infection by CD8^+^ T cells may be unrealistic, a T cell-based vaccine might limit early viral dissemination and immune damage by controlling HIV more effectively during initial infection, thereby leading to slower progression to AIDS [10].

Although there is evidence that CD8^+^ T cells control HIV, the mechanism by which these cells can do so is still poorly understood. Consequently, it is not known what functions will be required of vaccine-induced CD8^+^ T cells to protect against progression to AIDS. At present, virus-specific CD8^+^ T cells are mostly quantified using the IFNγ ELISpot assay when evaluating potential therapeutic and prophylactic vaccines [11], [12]. IFNγ is a well known anti-viral cytokine, which is readily produced and secreted by CD8^+^ T cells upon recognition of viral peptides presented on HLA class I molecules. It has been shown that IFNγ production by HIV-specific CD8^+^ T cells correlates with tetramer staining (which visualises the presence of specific T cells regardless of their function) and anti-viral cytotoxicity as measured by chromium release assays [11]. A high number of HIV-specific IFNγ producing CD8^+^ T cells has been associated with low viral load and sustained CD4^+^ T-cell counts [13]–[15]. Studies in HIV-infected individuals who seem able to control viremia without treatment have shown that these individuals have maintained highly functional T cells. Their HIV-specific CD8^+^ T cells are able to produce multiple cytokines (i.e. IFNγ, IL-2, TNFα) and are able to proliferate and degranulate upon in vitro peptide stimulation [13]. Production of IL-2 by CD8^+^ T cells is necessary for proliferation and differentiation into effector T cells. In contrast to long-term non-progressors, central memory CD8^+^ T cells capable of producing IL-2 are only rarely detected in viremic individuals progressing rapidly to AIDS [13], suggesting that IL-2 production plays an important role in CD8^+^ T cell function. Interestingly Gag, but not Env, specific responses were reported to correlate with low viral load [16], [17].

Cross-sectional studies have shown a striking relation between functional CD8^+^ T cells and low viremia [13], [14], [18], although others did not find such an association [19], [20]. Longitudinal studies performed in limited numbers of patients indicate however that in most untreated HIV-infected individuals, progression to AIDS occurred despite the presence of broadly directed cytokine-producing HIV-specific CD8^+^ T cells early in infection, suggesting that these cells are deleted or become functionally impaired as HIV infection progresses [21], [22]. Based on these data we have proposed that exhaustion of early HIV specific CTL because of chronic T cell activation and turnover may be directly related to disease progression [23].

Studies evaluating the role of CD8^+^ T cells in HIV infection thus far are often limited by small numbers of patients and/or a cross-sectional nature. To reveal the effect of HIV-specific CD8^+^ T cells on progression to AIDS, we therefore investigated in a large prospective cohort study whether the frequency of HIV-specific cytokine-producing CD8^+^ T cells approximately one year after seroconversion, when the viral set point has been established [24], [25], has a prognostic value for the rate of HIV-disease progression.

## Materials and Methods

### Patients

Ninety-six participants of the Amsterdam Cohort Studies on HIV-1 infection and AIDS with a known date of seroconversion were selected based on availability of cryopreserved PBMC approximately one year after seroconversion (median 12.3 months, range 0.3–20.4). Follow-up time was censored at July 1^st^ 1996, ensuring that none of the subjects received highly active anti-retroviral therapy (HAART). Median follow-up to AIDS diagnosis or censoring was 82.7 months (range 14.9–153.9). Forty of the 96 participants (42%) progressed to AIDS during follow-up. Details on inclusion criteria, enrolment and baseline characteristics of ACS participants can be obtained at www.amsterdamcohortstudies.org. Informed written consent was obtained from all participants and the study was approved by the Medical Ethical Committee of the Academic Medical Center.

### Intracellular cytokine staining after antigenic stimulation

Intracellular cytokine staining was performed as previously described [26]. In short, cryopreserved PBMC were thawed, aliquoted at 2×10^6^ cells per ml in round bottom tubes and stimulated with a Gag-peptide pool (15mers with 11 overlap, final concentration of the individual peptides was 2 µg/ml, HXB2, NIH AIDS Research and Reagent program, Bethesda, Maryland, United States) in the presence of co-stimulation (2 µg/ml αCD28 (Sanquin Reagents, Amsterdam, The Netherlands) and 2 µg/ml αCD49d (Pharmingen, San José, California, United States)) for 6 hours at 37°C, 5% CO2. As a positive control, cells were stimulated with a combination of PMA and Ionomycin (Sigma-Aldrich, Zwijndrecht, The Netherlands). After one hour, Brefeldin A (Becton Dickinson (BD), San José, California, United States) was added. After fixation and permeabilisation (permeabilisation reagents, BD) cells were stained for 20 minutes at 4°C with αCD3-PerCP, αCD4-APC, αIL-2-PE and αIFNγ-FITC monoclonal antibodies (BD), after which cells were fixed in Cellfix (BD) and flow cytometry was performed. First, lymphocytes were gated based on forward scatter and side scatter. Next, cytokine production (IFNγ, IL-2) was measured in CD3^+^ CD4^−^ T cells. At least 300.000 events were acquired and the data was analysed using the software program CELL Quest (BD). Frequencies of IFNγ and IL-2 producing cells were reported after subtraction of the frequencies in medium controls. Absolute numbers per volume blood were calculated by multiplication of the fraction of cytokine-positive CD8^+^ T cells with the absolute CD8^+^ T-cell count per microliter of blood.

### Statistical analyses

The effect of HIV-specific cytokine-producing CD8^+^ T cells for progression to AIDS was tested using Cox proportional hazards analyses and Kaplan-Meier survival curves. Individual markers were first tested univariately. In multivariate analyses, each marker was adjusted for plasma HIV-1 RNA and CD4^+^ T-cell counts [27]. Additionally we adjusted for Ki67 or CD38 expression by CD4^+^ T cells, or co-expression of CD38 and HLA-DR on CD8^+^ T cells, all markers known to be predictive for progression to AIDS [28]. Statistical analyses were performed using SPSS 12.0.1 (SPSS Inc, United States) and R 2.5.1 (R Development Core Team (2007), Austria).

## Results

### Patient characteristics

In this prospective cohort study, 96 participants of the Amsterdam Cohort Studies on HIV-1 infection and AIDS (ACS) with a known date of HIV-1 seroconversion were included. Since we aimed to investigate the potential prognostic effect of functional CD8^+^ T cells early after HIV infection, we analysed the capability of Gag-specific CD8^+^ T cells to produce cytokines upon in vitro stimulation of T cells sampled approximately one year after seroconversion in each individual (median 12.3 months post seroconversion, range 0.3–20.4 months post seroconversion), when the viral set point has been established. HLA class I typing was performed for all study participants. This revealed no selection of the HLA alleles known to be associated with either faster (e.g. HLA-B35) or slower (e.g. HLA-B27, HLA-B57/5801) progression to AIDS (Table 1). Detailed information on the distribution of HLA alleles in this cohort has been reported before [29]. Follow-up time was censored at July 1^st^ 1996, ensuring that none of the study participants received HAART. Median follow-up to AIDS diagnosis or censoring was 82.7 months (range 14.9–153.9 months). Forty of the 96 participants (42%) progressed to AIDS during follow-up. Table 1 shows an overview of the characteristics of our study population.

**Table 1:** Patient characteristics.

	Median (range)
Time of analysis (months after sc)	12,3 (0,3–20,4)
CD4 counts (cells/µl)	605 (260–1690)
CD8 counts (cells/µl)	730 (260–3640)
Plasma RNA load (copies/ml)	29.500 (400–310.000)
Follow-up until AIDS/censoring (months)	82,7 (15–154)
Frequency of HLA class I alleles:	
HLA-B35	16.9% (Caucasian population 18.6%)
HLA-B27	6.1% (Caucasian population 8%)
HLA-B57/5801	7.3% (Caucasian population 11%)
AIDS	40 (42%)

### Cytokine secretion profiles after stimulation with an overlapping Gag-peptide pool

**Figure 1:**
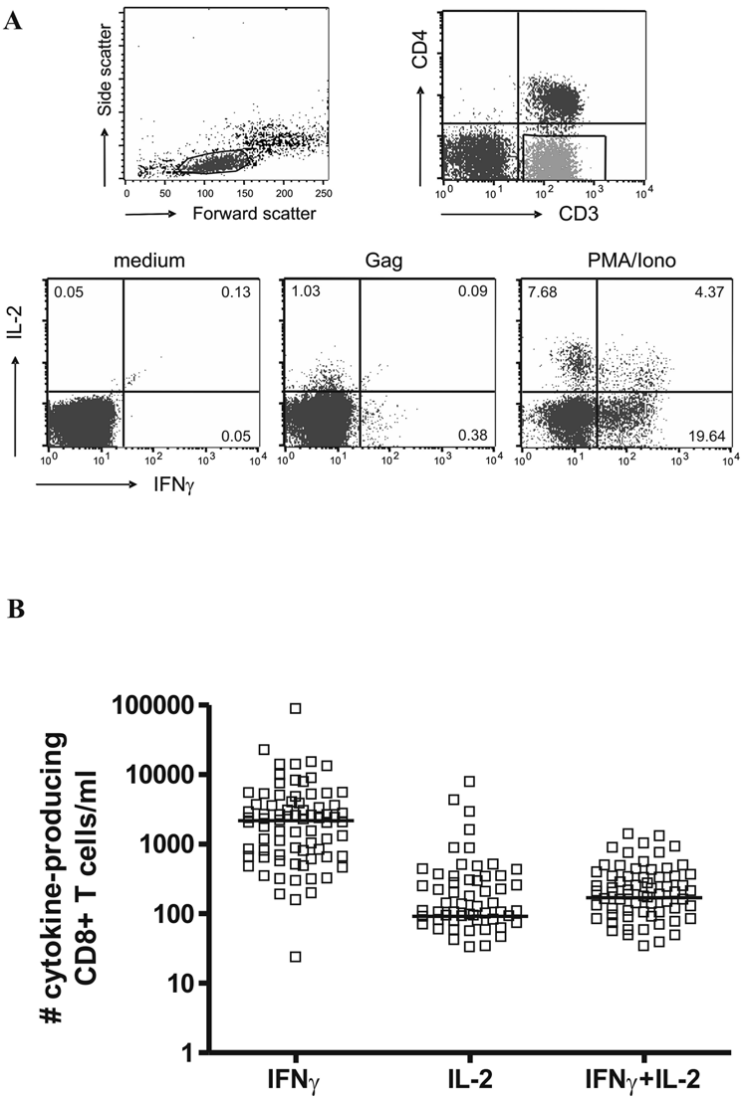
Cytokine secretion after stimulation with the overlapping Gag-peptide pool. (A) a representative FACS staining example. (B) an overview of the cytokine secretion profiles for the total study population. Each dot represents a single individual and the median is shown as a bar.

To test if the presence of HIV-specific cytokine-producing CD8^+^ T cells approximately one year after seroconversion was associated with AIDS-free survival, we measured the number and percentage of IFNγ and/or IL-2 secreting CD8^+^ T cells after in vitro stimulation with an overlapping Gag-peptide pool (Figure 1A). We examined IL-2 production because its autocrine production is especially important for CD8^+^ T cell responses in the setting of diminished CD4^+^ T-cell help [30], and IFNγ because it is the last cytokine to be lost during chronic antigen stimulation [31], and because IFNγ production is the primary read-out of immunity induced by T cell based vaccines currently under study [32]. One year after seroconversion, CD8^+^ T cells capable of secreting cytokines after stimulation with the Gag-peptide pool could be detected in almost all individuals, although at greatly varying levels. As can be seen from Figure 1B, HIV-specific CD8^+^ T cells predominantly secrete IFNγ, but also IL-2 production is frequently observed in our cohort early after HIV infection. The range of responses observed (IFNγ range 0–2.27%, mean 0.41%; IL-2 range 0–0.98%, mean 0.05%; IFNγ+IL-2 range 0–0.20%, mean 0.04%) were comparable to previously reported results by us and others in treatment-naïve patients [13], [30], [33].

**Figure 2:**
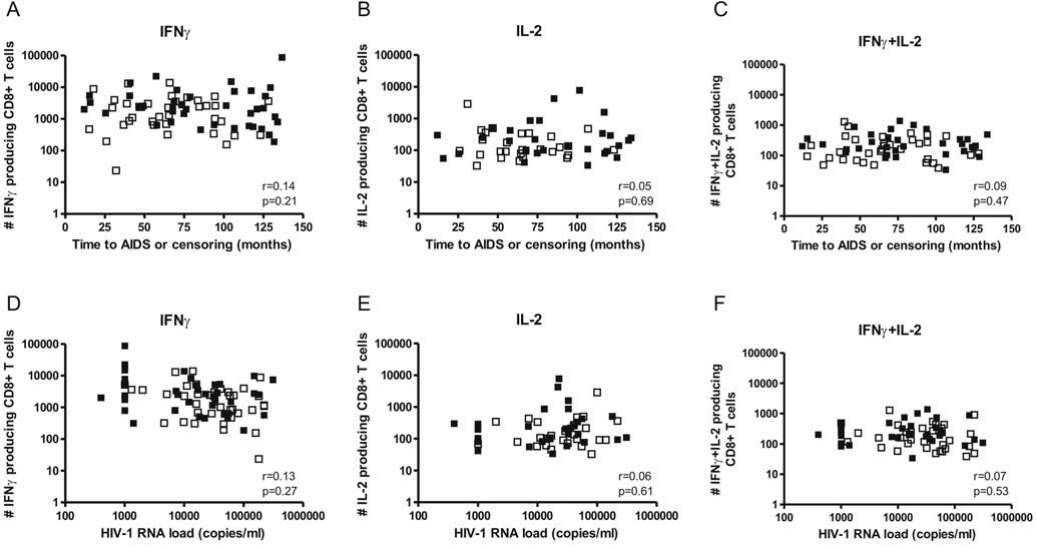
No correlation between cytokine producing CD8+ T cells and AIDS-free survival time or set point HIV RNA load. Numbers of IFNγ^+^ (A), IL-2^+^ (B) and IFNγ^+^IL-2^+^ (C) Gag-specific CD8^+^ T cells were plotted against the follow-up time in months for each study participant. Furthermore, numbers of IFNγ^+^ (D), IL-2^+^ (E) and IFNγ^+^IL-2^+^ (F) Gag-specific CD8^+^ T cells were plotted against the set point HIV RNA load for each study participant. No difference was observed between individuals followed until AIDS diagnosis (open squares) and censored individuals (closed squares). Correlations were calculated using Pearson correlation tests.

To investigate a possible correlation between in vitro cytokine production and AIDS-free survival time, the frequency of single IFNγ producing (Fig. 2A), single IL-2 producing (Fig. 2B) and double IFNγ+IL-2 producing (Fig. 2C) Gag-specific CD8^+^ T cells was plotted against the AIDS-free survival time of the study participants. No correlation could be found for any of the three cytokine profiles with AIDS-free survival time (all p-values >0.05, Pearson correlation test). Furthermore, no correlation was observed between IL-2 and/or IFNγ producing Gag-specific CD8^+^ T cells and set point HIV RNA load (Fig. 2D–F).

### No prognostic value of HIV-specific CD8^+^ T cells for AIDS-free survival

**Figure 3:**
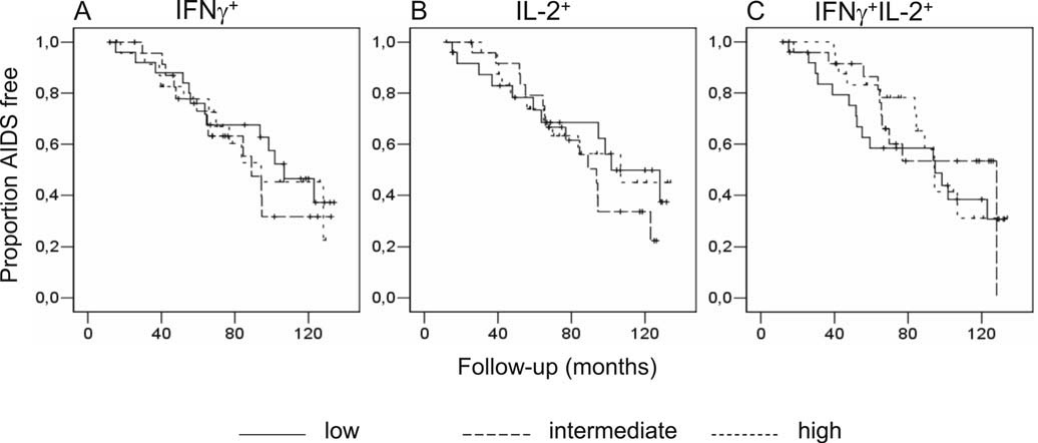
Gag-specific cytokine-producing CD8^+^ T cells have no prognostic value for progression to AIDS. Numbers of IFNγ^+^ (A), IL-2^+^ (B) and IFNγ^+^IL-2^+^ (C) Gag-specific CD8^+^ T cells were categorized in three groups corresponding to the level of production of the specific cytokine (low, intermediate or high) relative to the overall study population and analyzed univariately using Kaplan-Meier survival curves. Similar results were obtained using percentages of cytokine-producing CD8^+^ T cells instead of absolute numbers to circumvent potential fluctuations in CD8^+^ T-cell counts (data not shown).

To investigate the potential prognostic value of cytokine-producing CD8^+^ T cells for AIDS-free survival more accurately we utilized Kaplan-Meier survival curves. For all three cytokine profiles (e.g. single IFNγ, single IL-2 and IFNγ+IL-2) we first categorized each study participant in one of three equally-sized groups corresponding to the level of production of the specific cytokine (low, intermediate or high) relative to the overall study population. One year after seroconversion, neither the absolute number of single IFNγ producing (Fig. 3A), nor of single IL-2 producing (Fig. 3B) nor of double IFNγ+IL-2 producing (Fig. 3C) Gag-specific CD8^+^ T cells were significantly associated with AIDS-free survival time. Similar results were obtained using percentages of cytokine-producing CD8^+^ T cells instead of absolute numbers to correct for potential fluctuations in CD8^+^ T-cell counts (data not shown).

To exclude the possibility that factors known to be predictive for progression to AIDS might mask an effect of cytokine-producing HIV-specific CD8^+^ T cells, we also performed multivariate analyses using Cox proportional hazard analysis. When adjusted, respectively, for HIV-1 RNA load, total CD4^+^ T-cell counts, Ki67 expression on CD4^+^ T cells, and/or the activation status of CD4^+^ or CD8^+^ T cells, high numbers of cytokine-producing Gag-specific CD8^+^ T cells one year after seroconversion were again not associated with slow disease progression (Table 2).

**Table 2:** Cox Proportional Hazard analyses of cytokine-producing HIV-specific CD8^+^ T cells.

		Hazard ratio (95% CI)				
	# Cases^a^	Univariate	Multivariate 1^b^ adjusted	Multivariate 2^c^ adjusted	Multivariate 3^d^ adjusted	Multivariate 4^e^ adjusted
**# Gag IFNγ^+^ CD8^+^ T cells/ml** f						
**Low**	26	1	1	1	1	1
**Intermediate**	26	1,41 (0,648–3,069)	1,48 (0,674–3,259)	1,42 (0,635–3,166)	1,48 (0,672–3,251)	1,56 (0,701–3,474)
**High**	25	1,21 (0,539–2,705)	1,35 (0,593–3,052)	1,28 (0,553–2,952)	1,24 (0,539–2,863)	1,31 (0,570–2,990)
**# Gag IL-2^+^ CD8^+^ T cells/ml** g						
**Low**	26	1	1	1	1	1
**Intermediate**	27	1,52 (0,707–3,250)	1,49 (0,694–3,181)	1,45 (0,664–3,160)	1,43 (0,660–3,108)	1,36 (0,623–2,947)
**High**	26	1,00 (0,429–2,330)	1,02 (0,437–2,397)	1,05 (0,446–2,449)	1,08 (0,457–2,532)	0,97 (0,410–2,279)
**# Gag IFNγ^+^&IL-2^+^ CD8^+^ T cells/ml** h						
**Low**	25	1	1	1	1	1
**Intermediate**	25	0,76 (0,341–1,710)	0,85 (0,373–1,910)	0,86 (0,378–1,956)	0,85 (0,375–1,916)	0,81 (0,358–1,853)
**High**	25	0,81 (0,369–1,784)	0,92 (0,400–2,125)	0,90 (0,387–2,093)	0,98 (0,419–2,289)	0,88 (0,380–2,057)

a Because of missing data on the covariates of the multivariate model it is possible that a subject was excluded from the analyses.

b Adjusted for viral load (continuous) and CD4**^+^** T-cell count (continuous).

c Adjusted for viral load (continuous), CD4**^+^** T-cell count (continuous) and %Ki67**^+^**CD4**^+^** T cells (2 categories).

d Adjusted for viral load (continuous), CD4**^+^** T-cell count (continuous) and %CD38**^+^**CD4**^+^** T cells (2 categories).

e Adjusted for viral load (continuous), CD4**^+^** T-cell count (continuous) and %CD38**^+^**HLA-DR**^+^**CD8**^+^** T cells (2 categories).

f Ranges: low 0–840 cells/ml, intermediate 872–2886 cells/ml, high 3016–87724 cells/ml.

g Ranges: low 0–58 cells/ml, intermediate 58–141 cells/ml, high 177–7840 cells/ml.

h Ranges: low 0–104 cells/ml, intermediate 104–248 cells/ml, high 250–1387 cells/ml.

Alternatively we analyzed CD4^+^ T-cell decline as a surrogate marker for HIV-disease progression, via a random effects model for repeated measurements. This confirmed the finding that CD8^+^ T-cell responses measured approximately one year after seroconversion are not associated with the time to AIDS (data not shown).

Finally, we analyzed whether a combination of relatively high CD4^+^ and high CD8^+^ T-cell responses one year after seroconversion was associated with slow progression to AIDS. This revealed that individuals whose CD4^+^ *and* CD8^+^ Gag-specific T-cell response were in the highest tertile had a tendency for prolonged AIDS-free survival. This result was not significant, however, which might be due to the low number of individuals who met these criteria (n = 11, data not shown).

## Discussion

It has been suggested that strong CTL responses might be capable of controlling HIV replication in individuals with a long-term non-progressive disease course. In the present study we did not find any prognostic value of cytokine-producing HIV-specific CD8^+^ T cells measured approximately one year after seroconversion for progression towards AIDS, although at least two previous studies have suggested that high frequencies of HIV-specific T cells are associated with subsequent long-term non-progression [34], [35]. These studies were, however, based on very small numbers of patients and focused on only a limited number of HIV-1 epitopes. Moreover, the prospective approach taken in the current study has the advantage of little or no selection bias since it comprised all seroconvertors of the ACS and involved the largest patient group reported in prospective natural history studies thus far.

We quantified Gag-specific CD8^+^ T cells to investigate their prognostic value for AIDS-free survival. The individuals included in this study were infected in the late nineteen-eighties in Amsterdam, The Netherlands, therefore, we decided to use an overlapping peptide pool based on Gag sequences from the in 1983 isolated reference strain HXB2. There is always a risk of missing responses when using peptides that are not based on the autologous sequence. However, it has been shown that a large proportion of all T cell responses detected using autologous peptides was detected using consensus sequences as well [36]. Although one could argue that different results might have been found if peptide pools derived from other proteins of HIV had been used, we think this is not very likely. An overwhelming amount of data has suggested that Gag-specific CD8^+^ T-cell responses are most important in containing HIV infection. Kiepiela *et al* have shown a clear negative correlation between the number of epitopes targeted in Gag and HIV viral load, which was not seen for other HIV proteins [16]. The likelihood of progression to AIDS has been shown to be largest in patients who lack Gag-specific CTL [17], and the dominant CTL epitopes targeted in individuals with HLA alleles associated with slow disease progression (i.e. HLA-B27 and HLA-B57) have been shown to be derived from Gag [37]–[39]. Recently, Borghans *et al* have shown that the latter HLA alleles have an intrinsic preference to present Gag P24-derived epitopes, in contrast to other HLA alleles not associated with low viral load and slow disease progression [40]. Thus, Gag-specific CD8^+^ T-cell responses are implicated and would be the first to show an effect. Even using this potentially important protein we did not observe an association with AIDS-free survival. We cannot rule out the possibility, however that, in the early phase of chronic HIV infection, responses to early HIV-1 proteins dominate and might in part determine viral set point [41].

One could argue that measuring CD8^+^ T-cell responses one year after seroconversion might not be early enough to reveal a possible cause-effect relationship between HIV-specific cellular immunity and disease progression. However, since i) at that time all individuals have established their viral load set point and the immune system has largely rebounded from the initial effects of acute infection, ii) measuring CD8^+^ T cell responses too early after seroconversion might lead to false negative responses since it has been shown that the magnitude of the HIV-specific CD8^+^ T cell response is much lower in primary infection than in the chronic phase [42], iii) immunization of nonhuman primates with T-cell inducing vaccines resulted in a reduction of the viral load set point and delayed disease progression after SIV challenge [32], [43], and iv) almost all evidence for cellular protective immunity and correlation with viral load was obtained much later in infection, we believe that one year after seroconversion is the most reliable time point to reveal the cause-effect relationship between HIV-specific cellular immunity and disease progression if it exists. Although most individuals have been sampled between 8 and 16 months after infection, the range of sampling goes from as early as one month after infection till as late as twenty months after infection in our cohort. When we plotted the time after seroconversion that we sampled the individual patients against the number of HIV-specific CD8^+^ T cell capable of secreting cytokines (either IFNγ, IL-2, or both), we did not find any correlation what so ever (all p-values >0.17, Pearson correlation test, data not shown). Therefore, it appears that the exact time of sampling after seroconversion was not critical.

Our finding that the frequency of cytokine-producing HIV-specific CD8^+^ T cells one year after seroconversion has no prognostic value for the rate of disease progression does not imply that HIV-specific CD8^+^ T cells do not contribute to control during HIV infection. Their role in controlling viral load in both acute and chronic HIV infection has been firmly established [44]–[46]. Furthermore, rapid progression towards AIDS has been observed when CTL escape mutations arise in the HLA-B27 restricted KK10 epitope in Gag, highlighting the immune pressure CD8^+^ T cells can have on the virus [47], [48]. Our present study shows, however, that regardless of subsequent clinical outcome, high frequencies of cytokine-producing CD8^+^ T cells can be found shortly after seroconversion.

We have previously reported a lack of prognostic value of the number of HIV-specific CD4^+^ T cells producing IFNγ, IL-2, or both, one year after seroconversion [49]. It might be that individuals whose CD4^+^ *and* CD8^+^ Gag-specific T-cell response are particularly high have a tendency for longer AIDS-free survival. In the present study this holds true for only a small fraction of HIV-infected individuals, so it cannot explain the wide spectrum in HIV disease progression rates observed in untreated HIV infection. If neither CD4^+^ nor CD8^+^ HIV-specific T cells are associated with the rate of HIV-disease progression, the question remains what distinguishes individuals who progress rapidly or slowly to AIDS, and why typical progressors lose their functional HIV-specific CD8^+^ T cells faster compared to individuals with a long-term non-progressive disease course. It cannot be ruled out that other functions of CD8^+^ T cells than IFNγ or IL-2 production [13] or differences in avidity of the CD8^+^ T-cell repertoire may determine the clinical outcome of HIV infection. Since both progressors and long-term non-progressors have abundant T cell immunity of the type associated with low viral load early in infection, the more rapid loss of T cell immunity observed in progressors may be a consequence rather than a cause of disease progression, for which systemic immune activation might be a likely candidate [50]. Systemic immune activation may gradually exhaust memory and naive T cells, both HIV-specific as well as non-HIV-specific. This is in agreement with the finding that the set point immune activation level has been shown to predict progression to AIDS independently of viral load [28], [51]. Non-immune mechanisms have also been reported, such as expression of a human endogenous retroviral element recently found to be in linkage disequilibrium with HLA-B5701, the HLA class I allele most strongly associated with slow disease progression [52].

Recently, it became apparent that a CD8^+^ T cell-inducing vaccine has failed in a phase IIB clinical trial [53]. The trial was suspended after interim analysis had shown that the vaccine did not protect the trial participants against HIV infection, and even more important, participants who had received the vaccine and became HIV infected did not have a lower viral load set-point despite having robust CD8^+^ T-cell responses to the vaccine components (Gag, Nef and Pol) after vaccination. Although disappointing, these data are in line with our finding that the presence of high numbers of cytokine-producing HIV-specific CD8^+^ T cells does not guarantee a better clinical outcome.
